# β-Defensin 1 Is Prominent in the Liver and Induced During Cholestasis by Bilirubin and Bile Acids *via* Farnesoid X Receptor and Constitutive Androstane Receptor

**DOI:** 10.3389/fimmu.2018.01735

**Published:** 2018-07-27

**Authors:** Thomas Klag, Maria Thomas, Dirk Ehmann, Lioba Courth, Daniela Mailänder-Sanchez, Thomas S. Weiss, Rania Dayoub, Kerstin Abshagen, Brigitte Vollmar, Wolfgang E. Thasler, Eduard F. Stange, Christoph P. Berg, Nisar P. Malek, Ulrich M. Zanger, Jan Wehkamp

**Affiliations:** ^1^Department of Internal Medicine I, University of Tübingen, Tübingen, Germany; ^2^Dr. Margarete Fischer-Bosch Institute for Clinical Pharmacology, Stuttgart and University of Tuebingen, Tuebingen, Germany; ^3^University Children Hospital (KUNO), Regensburg University Hospital, Regensburg, Germany; ^4^Rudolf-Zenker-Institute for Experimental Surgery, University Medicine Rostock, Rostock, Germany; ^5^Department of Surgery, Grosshadern Hospital, Ludwig-Maximilians-University Munich, Munich, Germany

**Keywords:** cholestasis, antimicrobial peptides, human β-defensin-1, hepatocytes, bilirubin

## Abstract

**Background & aims:**

Knowledge about innate antimicrobial defense of the liver is limited. We investigated hepatic expression and regulation of antimicrobial peptides with focus on the human beta defensin-1 (hBD-1).

**Methods:**

Radial diffusion assay was used to analyze antimicrobial activity of liver tissue. Different defensins including hBD-1 and its activator thioredoxin-1 (TXN) were analyzed in healthy and cholestatic liver samples by qPCR and immunostaining. Regulation of hBD-1 expression was studied *in vitro* and *in vivo* using bile duct-ligated mice. Regulation of hBD-1 *via* bilirubin and bile acids (BAs) was studied using siRNA.

**Results:**

We found strong antimicrobial activity of liver tissue against *Escherichia coli*. As a potential mediator of this antimicrobial activity we detected high expression of hBD-1 and TXN in hepatocytes, whereas other defensins were minimally expressed. Using a specific antibody for the reduced, antimicrobially active form of hBD-1 we found hBD-1 in co-localization with TXN within hepatocytes. hBD-1 was upregulated in cholestasis in a graded fashion. In cholestatic mice hepatic AMP expression (Defb-1 and Hamp) was enhanced. Bilirubin and BAs were able to induce hBD-1 in hepatic cell cultures *in vitro*. Treatment with siRNA and/or agonists demonstrated that the farnesoid X receptor (FXR) mediates basal expression of hBD-1, whereas both constitutive androstane receptor (CAR) and FXR seem to be responsible for the induction of hBD-1 by bilirubin.

**Conclusion:**

hBD-1 is prominently expressed in hepatocytes. It is induced during cholestasis through bilirubin and BAs, mediated by CAR and especially FXR. Reduction by TXN activates hBD-1 to a potential key player in innate antimicrobial defense of the liver.

## Introduction

There is growing evidence that bacterial translocation from the gut may contribute to progression of chronic liver damage leading to liver fibrosis through increased inflammation (1–4). A compromised antibacterial barrier function in the gut, as prerequisite or consequence of liver cirrhosis, may, therefore, play a central role in its pathogenesis (1, 5, 6). On the other hand, once established, liver cirrhosis may lead to severe infectious complications like bacterial peritonitis and sepsis (7).

There are several mechanisms protecting the liver against the enormous variety of bacteria harbored in the intestine. First line of defense against these commensals and pathogenic microorganisms is the epithelium with its mucus barrier, consisting of mucins and antimicrobial peptides (AMPs) like alpha-defensins from Paneth cells in the small intestine and β-defensins in the colon, inhibiting bacterial translocation to portal blood (8). Human beta defensin-1 (hBD-1) is ubiquitous in most epithelia and in the colon, for example, it is regulated *via* nuclear receptors (NRs) like PPARγ (9). hBD-1 requires reducing conditions and oxidoreductases like thioredoxin-1 (TXN) or glutaredoxin-1 (GRX) (10) to turn on antimicrobial activity against several commensals as well as fungi (11).

The second line of defense against gut-derived microorganisms is inflammatory cells including granulocytes as well as lymphocytes and macrophages in the mucosa/submucosa and mesenteric lymph nodes. These bone marrow-derived cells may produce AMPs including defensins but also directly regulate antimicrobial peptide expression in Paneth cells *via* Wnt signaling (12). However, the liver is not only exposed to bacteria carried in portal vein blood (7) but also to microorganisms from the biliary tract, *via* ascending bacterial colonization from the duodenum. Nevertheless, bacterial colonization seems to be physiological and normally does not infect the healthy liver (13, 14).

Traditionally, the defense of the liver and its bile ducts against bacterial overgrowth was mainly attributed to bile salts, IgA secretion, mucus, and bile flow (15). However, studies of the past years have underlined the protective role of AMPs, since biliary epithelial expression has been found for AMPs like hepcidin, cathelicidin, and for the human β-defensins-1 and -2 (16–18). In addition, links to primary biliary cholangitis (PBC) and primary sclerosing cholangitis (PSC) were demonstrated, as there seem to be changes in biliary microbiota or diminished expression of AMPs, e.g., in PSC/PBC samples (13, 16, 18, 19).

Here, we aimed to systematically study the functional role of hepatic defensin expression, its activation, and regulation in the physiological situation and during cholestasis, because infectious complications are a common clinical problem in this condition (19, 20). At first we found a strong antimicrobial activity of liver tissue homogenates. After identification of hBD-1 as the quantitative key defensin in the liver we analyzed its known enzymatic activation system, potential physiological inducers during cholestasis like bilirubin and bile acids (BAs), and finally the molecular mechanisms (NRs) leading to induction of hBD-1.

## Materials and Methods

### Human Liver and Blood Samples

Liver tissue and corresponding blood samples were collected from a Caucasian patient cohort undergoing liver surgery (indications for surgery: benign or malignant liver tumors, liver abscess, caroli syndrome, or liver cysts) at the Department of Surgery, Campus Virchow, University Medical Center Charité, Humboldt University, Berlin, Germany as described before in Ref. (21, 22). All tissue samples had been examined by a pathologist, and only histologically normal liver tissue was used for further studies. 144 patients (69 males and 75 females; age: <20 years—2 patients; >20 to <70 years—116 patients; and >70 years—26 patients) were included. Patients who suffered from hepatitis, cirrhosis, or alcohol abuse were excluded. The study was approved by the ethics committees of the medical faculties of the Charité, Humboldt University and the University of Tuebingen, Germany and conducted in accordance with the Declaration of Helsinki. Written informed consent was obtained from each patient.

In addition, liver tissue from a second Caucasian patient cohort was collected from 138 patients undergoing liver surgery because of benign or malignant liver tumors at the Department of Surgery, University Medical Center Regensburg, Germany. Liver samples were scored by an intern pathologist regarding severeness of extrahepatic cholestasis: no cholestasis (*n* = 49), grade 1 (*n* = 26) mild (minor, low, and focal accentuated cholestatic alterations), grade 2 (*n* = 11) moderate (partly distinct cholestatic alterations), and grade 3 (*n* = 52) severe (pronounced, intense, strong, and extensive cholestatic alterations). The study was approved by the ethics committee of the University Medical Center Regensburg, Germany and conducted in accordance with the Declaration of Helsinki. Written informed consent was obtained from each patient.

#### Bile Duct-Ligated (BDL) Mice

All mice experiments were approved by the local government “Landesamt für Landwirtschaft, Lebensmittelsicherheit und Fischerei,” Mecklenburg-Vorpommern, Germany (LALLFM-V/TSD/7221.3-1.2-049/09) and performed in accordance with the German legislation on protection of animals and the National Institutes of “Health Guide for the Care and Use of Laboratory Animals” (Institute of Laboratory Animal Resources, National Research Council; NIH publication 86-23 revised 1985). Male C57BL/6J (Charles River Laboratories, Sulzfeld, Germany) at 8–10 weeks of age with a body weight of 23–26 g were kept on water and standard laboratory chow *ad libitum*. The detailed description of the surgical procedure and time-resolved sampling is described elsewhere in Ref. (23). Liver tissue at indicated time points post-surgery was partially embedded in paraffin for morphology analysis and snap frozen for molecular biology and biochemistry analyses. Gene expression analysis was performed as described below.

#### Protein Isolation of Human Liver Tissue and Immunoblot Analysis for hBD-1

Normal liver tissue was shred with a ball mill and proteins were extracted by incubation in 5% acetic acid containing 6.7 µM PMSF, 9.75 μMol pepstatin, and 15.7 µM leupeptin for 2 h on ice. After centrifugation the supernatant was lyophilized in a Speed-Vac (Modell) and resolved in 0.01% acetic acid containing 3.75 µM PMSF, 5.5 µM pepstatin, and 8.8 µM leupeptin. For cationic protein extracts we used a weak cation exchange matrix, as previously described in Ref. (24). For immunoblot analysis we used 1 and 2 µl protein extract onto a Amersham Protan 0.1 µm nitrocellulose membrane (GE Healthcare, Germany). After washing in PBST, we shook the membrane in PBST with 3% nonfat powdered milk for 1 h at room temperature. After this blocking step we incubate the blot in a 1:500 dilution of hBD1 antibody (0.1 mg/ml, Peprotech 500-P253, USA) at 4°C overnight. After three times of washing with PBST we used a 1:5,000 dilution of goat anti-rabbit HRP conjugated secondary antibody (Abcam ab6721, UK). For visualization we used the Super Signal West Dura Extended Duration Substrate (Thermo Scientific, USA). Images were made with the Chemi Doc Hi system (BioRad, USA). The study was approved by the ethics committee of the medical faculty of the University of Tuebingen, Germany and conducted in accordance with the Declaration of Helsinki. Written informed consent was obtained from each patient.

#### Radial Diffusion Assay (RDA)

Antimicrobial RDA was performed as described previously in Ref. (11), using instead of 2 mM DTT 2 mM TCEP as reducing agent. Briefly, logarithmic *Escherichia coli* MC1000 were washed and 4 × 10^6^ cfu/ml were used for killing assay. For the low-nutrient gel we used 10 mM sodium phosphate with 0.3 mg/ml trypticase soy broth and 1% (w/v) low EEO-agarose (Applichem, Germany) with 0–2 mM TCEP (Roth, Germany). For each protein extract, 8 µg was pipetted in punched wells (determind by Bradford) and a nutrient rich gel was poured onto the plates after 3 h incubation time at 37°C. After an overnight incubation at 37°C pictures were made to determine the inhibition zones.

#### Cell Culture and Treatments

The use of primary human hepatocytes (PHH) for research purposes was approved by the local ethics committees of the Ludwig-Maximilians-University of Munich, the Charité, Humboldt University Berlin and the University of Regensburg, and written informed consent was obtained from all patients. Hepatocytes from seven donors (see Table S1 in Supplementary Material) were isolated and cultured as described previously in Ref. (25). Detailed description of culturing HepaRG cells can be found elsewhere in Ref. (25, 26). Briefly, HepaRG cells (batch HPR101007) were obtained from Biopredic International (Rennes, France) and expanded according to the provider’s instructions. The cells were cultivated for the first 14 days in HepaRG growth medium based on William’s E Medium with supplements. At the final stage, HepaRG cells reached a differentiated hepatocyte-like morphology and showed liver-specific functions. All cells were maintained at 37°C and 5% CO_2_ in a humidified atmosphere throughout the experiments.

For the treatment experiments (HepaRG/PHH), 50 or 100 µM bilirubin or different concentrations of pooled BAs (both Sigma-Aldrich, Germany) dissolved in DMSO were added to the cell medium for 24 or 48 h prior assessment of RNA expression. 24 µM of bilirubin is an estimated equivalent of 1.4 mg/dl bilirubin in the human serum (27). The used doses with 50 and 100 µM of bilirubin (about 2.9–5.8 mg/dl estimated human serum equivalent) therefore represent the average bilirubin level in our cholestasis group in the 144 human liver tissue samples, see Section “Results.” A mixture of three BAs, chenodeoxycholic acid, lithocholic acid, and deoxycholic acid (Sigma-Aldrich, Germany) was used as “bile acids pool.” Cholic acid, which is known to have additional and differential functions, i.e., intercrossing with fatty acid metabolism was excluded due to anticipated broad spectrum of potential indirect effects.

Additional reagents were used in the performed induction experiments: 100 µM of PPARα agonist (WY14,643), 10 µM of farnesoid X receptor (FXR) agonist (GW4064), 10 µM Rosiglitazone (PPARγ agonist), 10 µM Rifampicin (PXR agonist), 10 µM TO-90 (LXRα agonist), 10 ng/ml of IL-6 or solvent control, DMSO (all purchased by Sigma-Aldrich, Germany), as well as 5 µM 6-(4-chlorophenyl)imidazo[2,1-b][1,3]thiazole-5-carbaldehyde-O-(3,4-dichlorobenzyl)-oxime (CITCO), (Enzo Life Sciences).

#### Transfections and Luciferase Reporter Analyses

HuH7 cells were transfected with the Firefly luciferase reporter construct, carrying hBD-1 promoter sequence upstream of the luciferase gene, using standard methods as recently described in Ref. (9, 28, 29). The plasmid pRL-CMV, encoding Renilla luciferase under the control of a constitutively active viral promoter, was co-transfected for normalization purposes. 24 h after seeding of the cells, 800 ng of plasmid DNA (750 ng of the respective Firefly luciferase reporter plasmid, 50 ng pRL-CMV) were transfected per well (24-well plate) using Lipofectamine 2000 (Invitrogen). Transfection experiments with the pGL3Basic empty vector were conducted as controls. Cells were incubated with indicated agonists or v/v DMSO control for 48 h prior to lysis with 1× passive Lysis Buffer (Promega) and luciferase activity determination as previously described in Ref (30).

#### Transfections With Small Interfering RNAs (siRNAs)

For RNA interference experiments, HepaRG cells were transfected with 20 nM siRNAs using 10 pM Lipofectamine RNAiMAX Transfection Reagent (Life Technologies) in 12-well plate with serum-free medium. The siRNA targeting PPARα, FXR (NR1H4; Life Technologies, 137883), constitutive androstane receptor (CAR) (NR1I3; Life Technologies, 137881), and a non-targeting siRNA as a negative control (Lo GC Duplex 2) were obtained from Life Technologies. 100 microliters of the transfection cocktail was added per well to the cells (100 µl culture medium). Upon 20 min of complex formation, the liposomes were given to the cells (29, 31).

#### Quantitative Real-Time PCR Gene Expression Analysis

For the determination of the absolute amounts of hBD-1, hBD-2, hBD-3, hBD-4, TXN, and GRX mRNA expression in the cohort of human liver samples (*n* = 144), high quality total RNA was isolated from liver tissue using Trizol/Qiagen RNeasy protocol as described previously in Ref. (32). Only high-quality total RNA (RNA integrity number > 7) was used. Synthesis of cDNA was performed with 1 µg RNA using the TaqMan Reverse Transcription Kit (Applied Biosystems) according to the supplier’s instructions. Measurements were performed using previously described quantitative gene expression assays with a LightCycler system (Roche Diagnostics) (10). Primers were used as described in Table S2 in Supplementary Material. Used primers for GRX and TXN were described before and standard curves for all measurements were obtained using cDNA-containing linearized plasmid DNA as described before in Ref. (10).

For the quantitative assessment of the hBD-1 gene expression in the lysates of PHH and HepaRG cells, as well as in the liver homogenates of the second patient cohort (*n* = 138), the established protocol for parallel analysis of gene expression using Fluidigm Biomark HD and Taqman Assay (Hs00174765_m1; Life Technologies) was performed (33). Raw data were normalized to RPLP0 (60S large ribosomal protein P0) expression which was determined in the same samples using the endogenous control assay (4326314E; Life Technologies).

Similarly, qPCR analysis of the liver homogenates of BDL mice was performed using Fluidigm Biomark HD system using mouse-specific assays: Mm00432803_m1 (Defb1), Mm04214158_s1 (Defb3), Mm04231240_s1 (Hamp), Mm00726847_s1 (Txn1), and Mm00728386_s1 (Glrx). The mRNA expression levels were normalized to GAPDH (Mm99999915_g1) expression. Relative gene expression changes were calculated using the (ΔΔCt) method (33).

#### Western Blot Detection of Protein Expression (siRNA Knock-Down Control)

For CAR knock-down validation we provide the mRNA expression levels of CYP2B6 as a target gene of CAR. PPARα and FXR protein expression levels following siRNA treatment in HepaRG cells were assessed by Western blot (see Figure S4 in Supplementary Material). Antibodies used: rabbit anti-human PPARα, (CAYMAN No. 101710, dilution 1:500), FXR (Abnova H00009971-M01, dilution 1:1,000), mouse anti-β-Actin (Sigma-Aldrich, A5441, 1:500), goat-anti-rabbit-IRD800 (Li-COR, 926-32214, 1:10,000), and goat-anti-mouse-IRD650 (Li-COR, 926-68074, 1:10,000) were used as fluorescently labeled secondary antibodies.

#### Immunofluorescence Staining and Immunohistochemistry

Paraffin-embedded samples were deparaffinized and rehydrated. Slides were incubated in preheated 0.1 M sodium citrate buffer pH 6.0 in a steamer for 20 min. For immunofluorescence analysis, sections were blocked with 10% donkey serum in PBS at room temperature for 30 min followed by incubation with rabbit antiserum to DEFB1 (Abgent 1:50 dilution) and goat antiserum to Thioredoxin (R&D Systems, 1:50 dilution) over night at 4°C. Donkey anti-rabbit-Cy3 and donkey anti-goat-Alexa 647 were used as secondary antibodies (both Dianova, diluted 1:500). Nuclei were stained with Yopro (Invitrogen, 1:2,000 dilution). All washing and antibody addition steps were performed with PBS + 0.05% BSA and Tween 20, respectively. The sections were analyzed with a confocal laser scanning microscope (Leica TCS SP; Leica Microsystems) at 400× magnification.

Immunohistochemistry experiments for detection of reduced hBD-1 were performed as described before in Ref. (10, 11). Dot-blot control of antibody specificity (reduced hBD-1) was performed as described previously in Ref. (11).

#### Statistical Analyses

For demonstration of gene expression changes, mean fold changes as obtained from the ΔΔCT-method and their SDs were calculated. To determine the significance of gene expression changes, grouped *t*-test with Bonferroni *post hoc* test for multiple comparisons or Mann–Whitney test was applied using GraphPad Prism 5.0.4 software (GraphPad Software, Inc., La Jolla, USA).

## Results

### Human Liver Tissue Has Strong Antimicrobial Function Accompanied by a High Expression of Human β-Defensin 1 and Oxidoreductases

**Figure 1:**
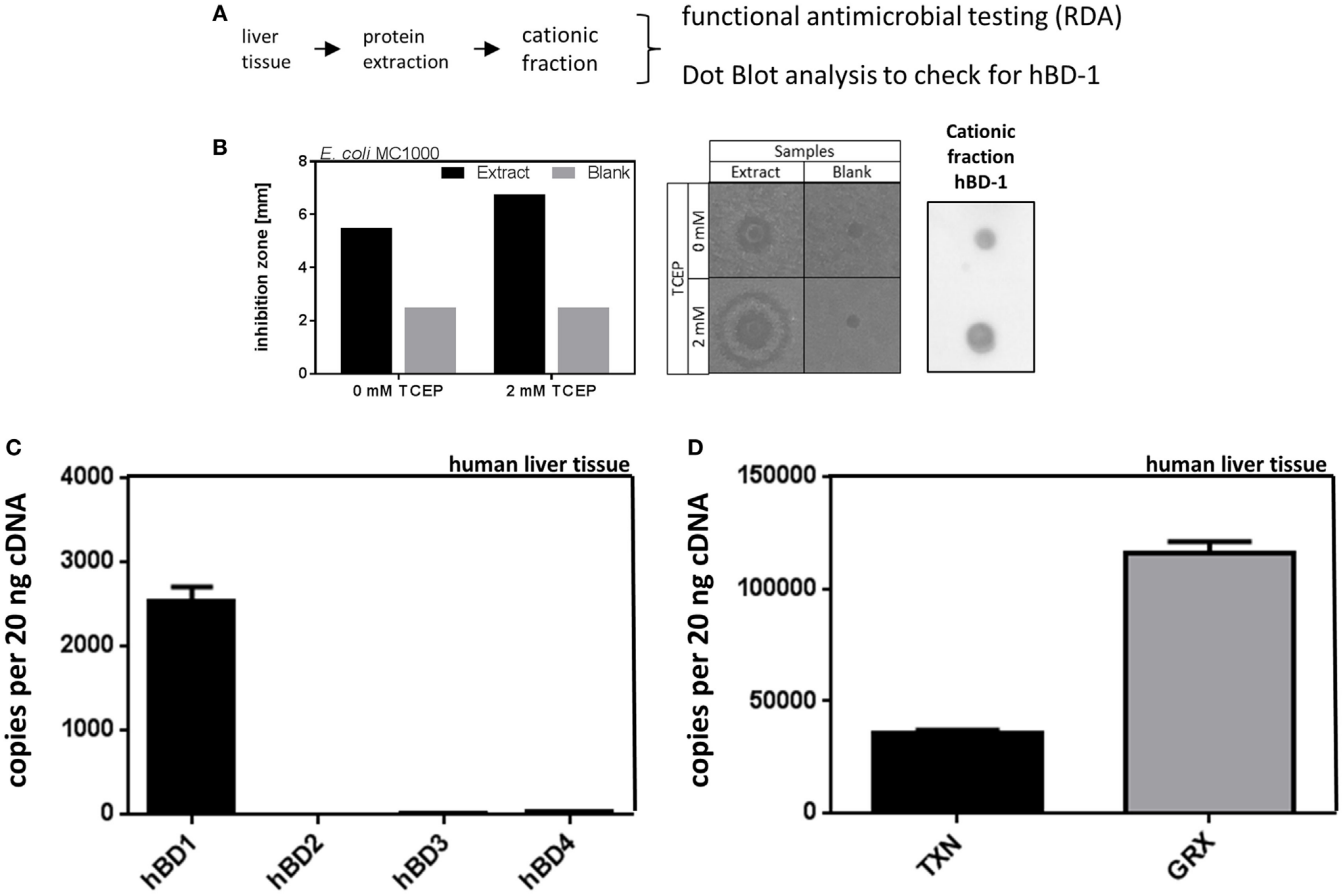
Antimicrobial activity of liver protein extract is accompanied by high expression of hBD-1 and oxidoreductases in human liver tissue. **(A)** Schematic workflow of cationic protein extraction from liver tissue, which was tested for antimicrobial activity [radial diffusion assay (RDA)] and hBD-1 status (by dot blot analysis). **(B)** On the left panel, quantitative results of RDA inhibition zones of cationic protein extract and blank extract (without liver tissue) under reducing (2 mM TCEP) or non-reducing conditions (0 mM TCEP) are depicted. In the middle panel, the respective RDA results are shown as picture. On the right panel, dot blot results with hBD-1 primary antibody to confirm hBD-1 peptide in cationic protein extract are shown. **(C)** mRNA expression of hBD-1, hBD-2, -3, and -4 was analyzed in a human patient cohort without cholestasis or systemic inflammatory response (*n* = 120). mRNA transcript levels are measured in an amount of 20 ng cDNA. Total copy numbers are depicted. Data are presented as means ± SEM for each gene assay. **(D)** mRNA expression of TXN and GRX in human liver tissue (*n* = 120). mRNA transcript levels are measured in an amount of 20 ng cDNA. Total copy numbers are depicted. Data are presented as means ± SEM.

In a first step we analyzed the antimicrobial effect of liver tissue against bacteria. For this end we performed RDAs checking for antimicrobial activity of liver tissue homogenates against the common gut microbe *E. coli*. We found a strong inhibition of *E. coli* growth, using cationic protein fractions of liver homogenates, where hBD-1 is cumulating, as shown by immunoblot analysis (Figure 1). Under reducing conditions, using 2 mM TCEP as reducing agent, we found an increased antimicrobial activity of the cationic fraction of liver homogenates. This is consistent with the known biochemical properties of hBD-1, which is more antimicrobial active when chemically reduced (11).

To further study β-defensins in general as potential mediators of the above described antimicrobial activity, we analyzed the mRNA expression of β-defensins and oxidoreductases in human liver specimens (*n* = 120). With about 2,500 copies per 20 ng cDNA we found constitutive expression of hBD-1 in the human liver as well as constitutive expression of thioredoxin (TXN) and glutaredoxin-1 (GRX) (Figures 1C,D). However, there was only minimal expression of hBD-2, hBD-3, or hBD-4 at a threshold of about 50 copies per 20 ng cDNA (Figure 1C). We also performed a screening study to investigate the basal expression of several defensins in primary cultured human hepatocytes (PHH) (Figure S1 in Supplementary Material) and again hBD-1 mRNA was prominent while other β-defensins were only marginally expressed or absent. Based on these data we focused on the expression and regulation of hBD-1 in the liver.

### hBD-1 and TXN Are Co-Localized in Human Liver Tissue

**Figure 2:**
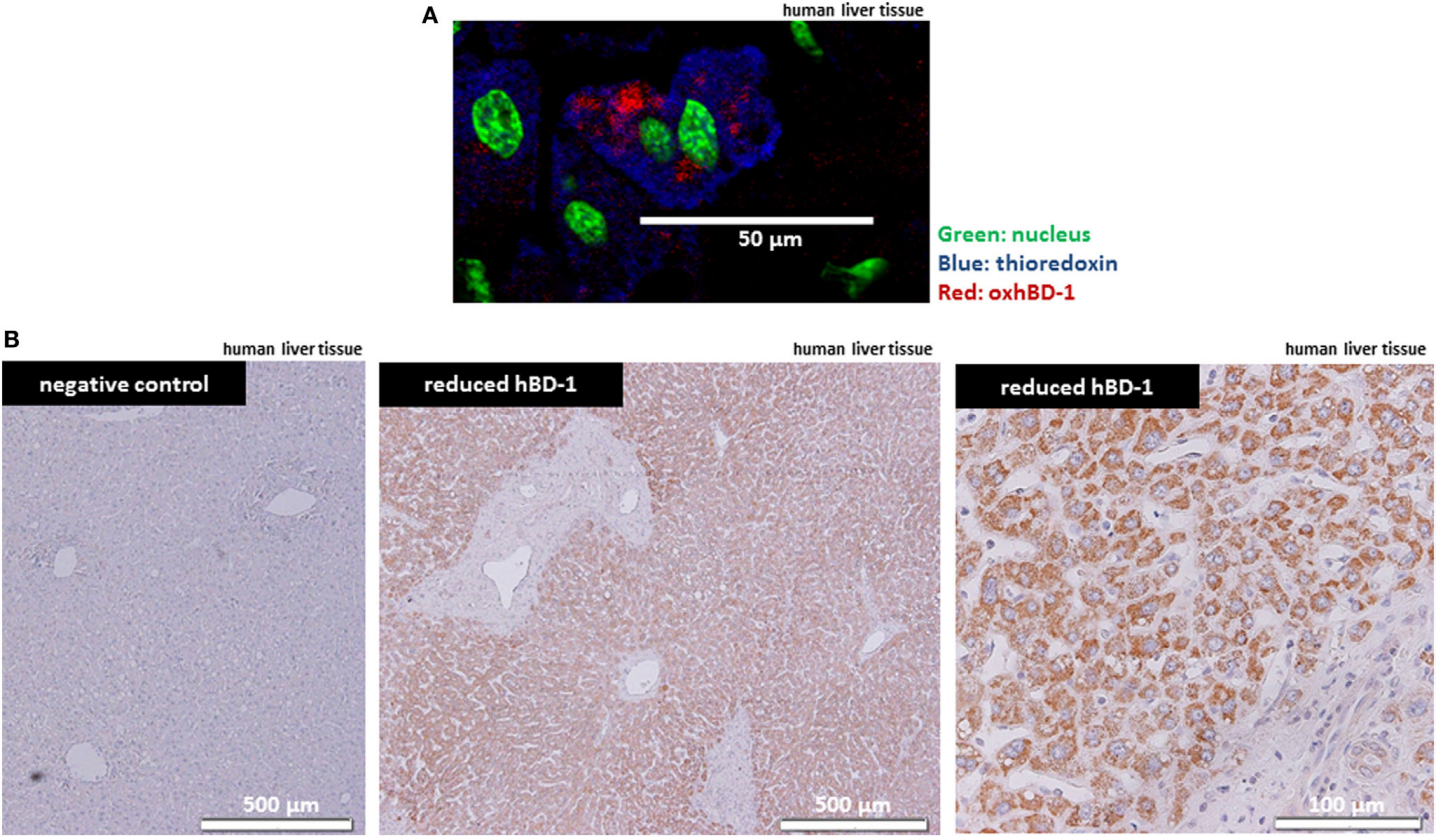
Antimicrobial active—reduced hBD-1—is strongly expressed in human hepatocytes. **(A)** Liver tissue from a representative healthy person was stained for TRX (blue) and oxidized hBD-1 (red), showing hBD-1 and TXN being co-localized in human hepatocytes. Nuclear staining is depicted in green. **(B)** Staining of reduced hBD-1 shows a strong and ubiquitous expression of reduced hBD-1 in human liver tissue.

Since hBD-1 antimicrobial activity is known to be activated through chemical reduction by TXN in the intestine (10), we studied the presence of hBD-1 and TXN in human liver tissue by immunofluorescence staining and immunohistochemistry. Here, we found a ubiquitous strong expression of TXN in human hepatocytes on the protein level, as well as a hepatocellular expression of oxidized hBD-1, co-localizing with TXN in hepatocytes (Figure 2A). This ubiquitous expression suggested that oxidized hBD-1 may be rapidly reduced by TXN in the liver and indeed when using an antibody specific for the reduced form of hBD-1 (redhBD-1) (for antibody characteristics see Figure S2 in Supplementary Material) we observed a ubiquitous and strong hepatocyte staining (Figure 2B).

### Induction of hBD-1 and TXN in Cholestasis

**Figure 3:**
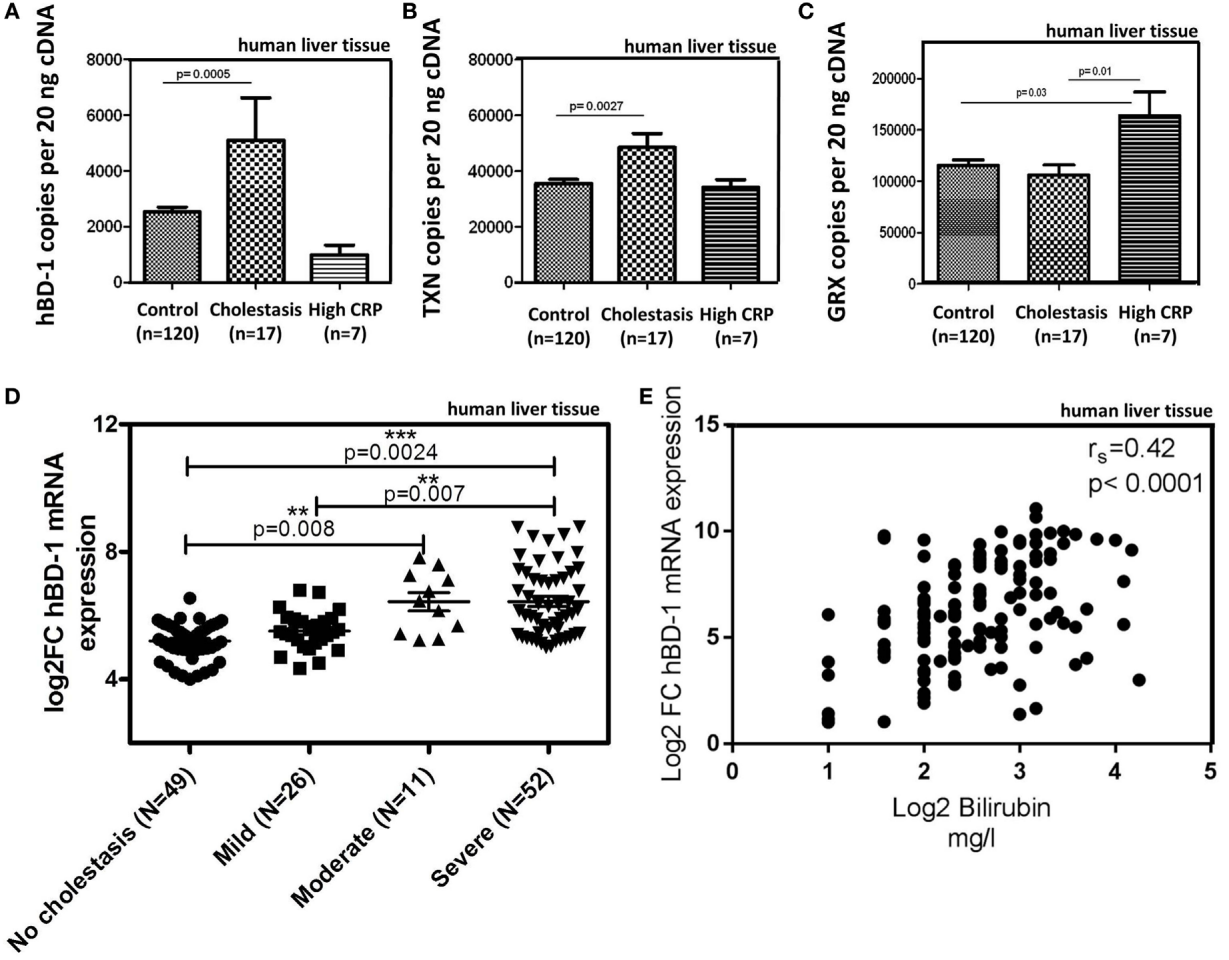
hBD-1 expression in samples of patients with cholestasis. **(A–C)** mRNA expression of hBD-1 **(A)** and human oxidoreductases TXN **(B)** and GRX **(C)** were analyzed in human liver tissue (*n* = 144). Control group (*n* = 120) without cholestasis was compared with cholestatic samples (serum bilirubin > 1.2 mg/dl, mean 4.5 mg/dl) and samples with signs of systemic inflammatory response (CRP > 5 mg/l). mRNA transcript levels are measured in an amount of 20 ng cDNA. Total copy numbers are depicted. Data are presented as means ± SEM. Values between groups were considered statistically significant with *p* < 0.05. **(D)** Hepatic hBD-1 mRNA expression was assessed in a cohort of patients with different grades of cholestasis (*n* = 138). Depicted are scatter blots of individual hBD-1 expression with horizontal lines indicating medians. Data were analyzed by Mann–Whitney test. **p < 0.01; ***p < 0.005. **(E)** Correlation matrix between mRNA expression of hBD-1 and bilirubin serum values. Spearman’s rank correlation coefficient is indicated as *r_s_*. Statistical significance for comparison was *p* < 0.0001.

Most interestingly, in the presence of cholestasis (in 17 of the 144 patient cohort, defined as serum bilirubin levels >1.2 mg/dl; mean 4.5 mg/dl) we found a significant induction of hBD-1 (about 2.5-fold) and TXN but not GRX (Figures 3A–C). Elevated serum C-reactive protein levels (>5 mg/l), as marker of a systemic inflammatory response, had no effect on hBD-1 and TXN expression (Figures 3A,B), but was associated with enhanced GRX (Figure 3C). Furthermore, we observed that in liver specimen from subjects with “mild cholestasis” defined as elevated serum cholestasis markers alkaline phosphatase (AP) and gamma-glutamyl transferase (GGT) [AP >147 units/l (female), >176 units/l (male); GGT >40 units/l (female), >60 units/l (male)] without elevation of serum bilirubin, hBD-1 (and GRX) expression was not induced, in contrast to significant induction of TXN (*p* < 0.01) (Figures S3A–C in Supplementary Material).

To explore whether hBD-1 mRNA expression is connected to progressive cholestasis in patients, we quantified the mRNA expression of hBD-1 transcripts in a further patient cohort (*n* = 138), comparing non-cholestatic tissue samples with samples of different severity of extrahepatic cholestasis based on histopathology. Patients were categorized into four groups: a control group with no signs of cholestasis (*n* = 49), mild (*n* = 26), moderate (*n* = 11), and severe cholestasis (*n* = 52). Indeed, hBD-1 expression increased stepwise with the degree of cholestasis (Figure 3D). Interestingly, expression levels of hBD-1 mRNA significantly correlated with bilirubin serum levels (Figure 3E), with a Spearman’s rank of *r* = 0.42 in this cohort (*p* < 0.0001).

### Cholestasis in BDL Mice Induces AMPs

**Figure 4:**
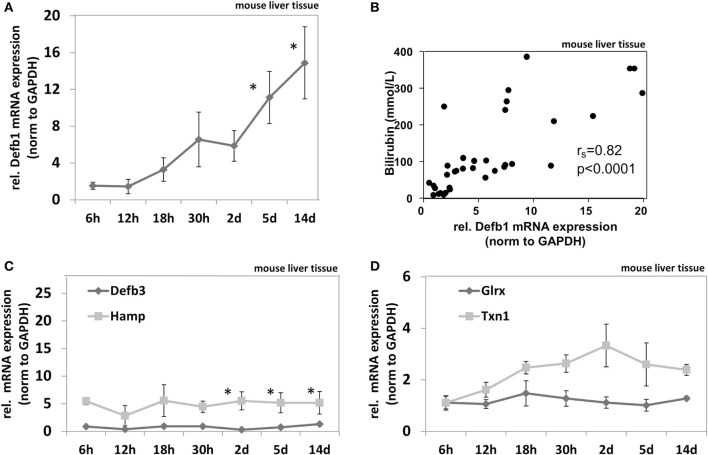
*In vivo* expression of murine antimicrobial peptides in liver homogenates of bile duct-ligated mice. Gene expression pattern of Defb1 **(A)**, Defb3 and Hamp **(C)**, as well as Glrx and Txn1 **(D)** at multiple time points after bile duct ligation. Gene expression levels are shown as fold changes to sham-operated mice (0 h), normalized to GAPDH levels as average of 5 mice per time point. Error bars indicate SDs. Data were analyzed by student’s *t*-test; *p < 0.01. **(B)** Correlation matrix between mRNA expression of Defb1 and bilirubin values. Spearman’s rank correlation coefficient is indicated as *r_s_*. Statistical significance for comparison was *p* < 0.0001.

To test if cholestasis-mediated induction of AMPs is a common biological feature we measured AMP mRNA expression in BDL mice. Detailed biochemical characteristics of the BDL mice cohort were described elsewhere in Ref. (23). BDL mice (*n* = 5 per group) were analyzed at different time points after bile duct ligation (6, 12, 18, and 30 h and days 2, 5, and 14). It was previously shown that bile duct ligation induced time-dependent progressive stages of cholestasis in these mice as well as elevation of serum bilirubin levels (23). Gene expression of murine AMPs including murine defensin beta 1 (Defb1), murine defensin beta 3 (Defb3) (homolog of the human beta defensin 2) (34), and murine hepcidin antimicrobial peptide (Hamp) as well as the murine oxidoreductases glutaredoxin (Glrx) and thioredoxin-1 (Txn1) was measured in liver homogenates of BDL mice at multiple time points after bile duct ligation compared to sham-operated mice (Figure 4). We found significantly enhanced beta defensin 1 mRNA expression in cholestasis (Figure 4A), again displaying a significant correlation to serum bilirubin levels (Spearman’s rank = 0.82; *p* < 0.0001) (Figure 4B). Measuring other AMPs in BDL mice revealed that Hamp but not Defb3 was also induced but stable over 2 weeks despite progressing cholestasis (Figure 4C). Furthermore, we found a numerical (not significant) induction of Txn1 after 2 days in BDL mice but no induction of Glrx (Figure 4D).

### Bilirubin and BAs Induce hBD-1 Expression in Human Hepatocyte Cultures

**Figure 5:**
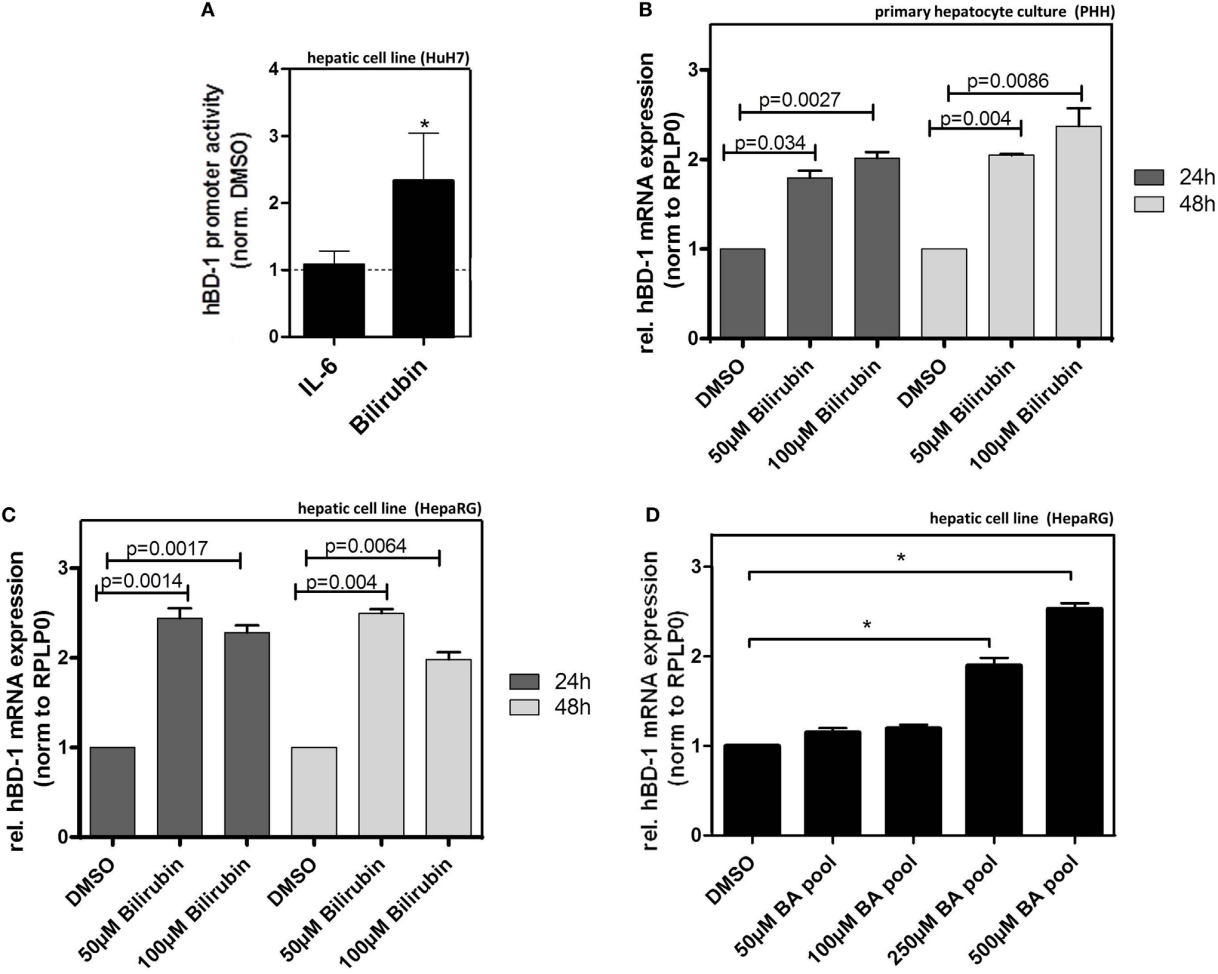
Bilirubin and bile acids (BA) induce expression of hBD-1 in hepatic cell lines. **(A)** Quantitative luciferase reporter gene assays were performed in HuH7 cells 48 h after treatments with 10 ng/ml IL-6 or 100 µM bilirubin in total 72 h after transfection with the luciferase-containing constructs. The cells were co-transfected with the vector containing Renilla luciferase for normalization of transfection efficiency. The bars represent average fold change lucifearse induction normalized to solvent, PBS (for IL-6), or DMSO (for bilirubin), control. Error bars indicate SD between three independent experiments. *p < 0.05. **(B)** qPCR analysis of hBD-1 expression in three independent batches of primary human hepatocytes, 24 or 48 h following indicated treatment. The bars represent average fold change expression levels normalized to hBD-1 expression in solvent, DMSO, and control. Error bars indicate SD between three independent experiments. **(C)** Similar to **(B)** experiments in HepaRG cells performed in two independent HepaRG cultures in two technical replicates. The significance was calculated using data generated in quadriplicates. **(D)** Treatment of HepaRG cells with different concentrations of BA pool. hBD-1 mRNA expression was assessed 48 h after treatment with the indicated BA concentrations. The bars represent average fold change expression levels normalized to solvent, DMSO, and control. Error bars indicate SD between three independent experiments. *p < 0.05.

Given the above results we hypothesized that bilirubin and/or BAs might be relevant inducers of hBD-1 during cholestasis. Treatment of HuH7 cells with bilirubin elicited a significant induction in the luciferase assay following transfection with an hBD-1 promoter-containing construct, an effect not seen with interleukin-6 (IL6) treatment (Figure 5A). To confirm these data for mRNA expression, we tested the effect of bilirubin on hBD-1 expression in two further hepatic cell systems. These data showed that treatment with bilirubin at different concentrations approximately doubled hBD-1 mRNA expression in cultures of PHH as well as HepaRG cells (Figures 5B,C). The effect was stable for 48 h. Similarly, treatment of HepaRG cells with pooled BAs also led to a significant induction of hBD-1 expression (Figure 5D) but in contrast to bilirubin this effect was dose dependent.

### Bilirubin- and Ligand-Mediated Activation of NRs Modulate hBD-1 Expression in HepaRG Cells

**Figure 6:**
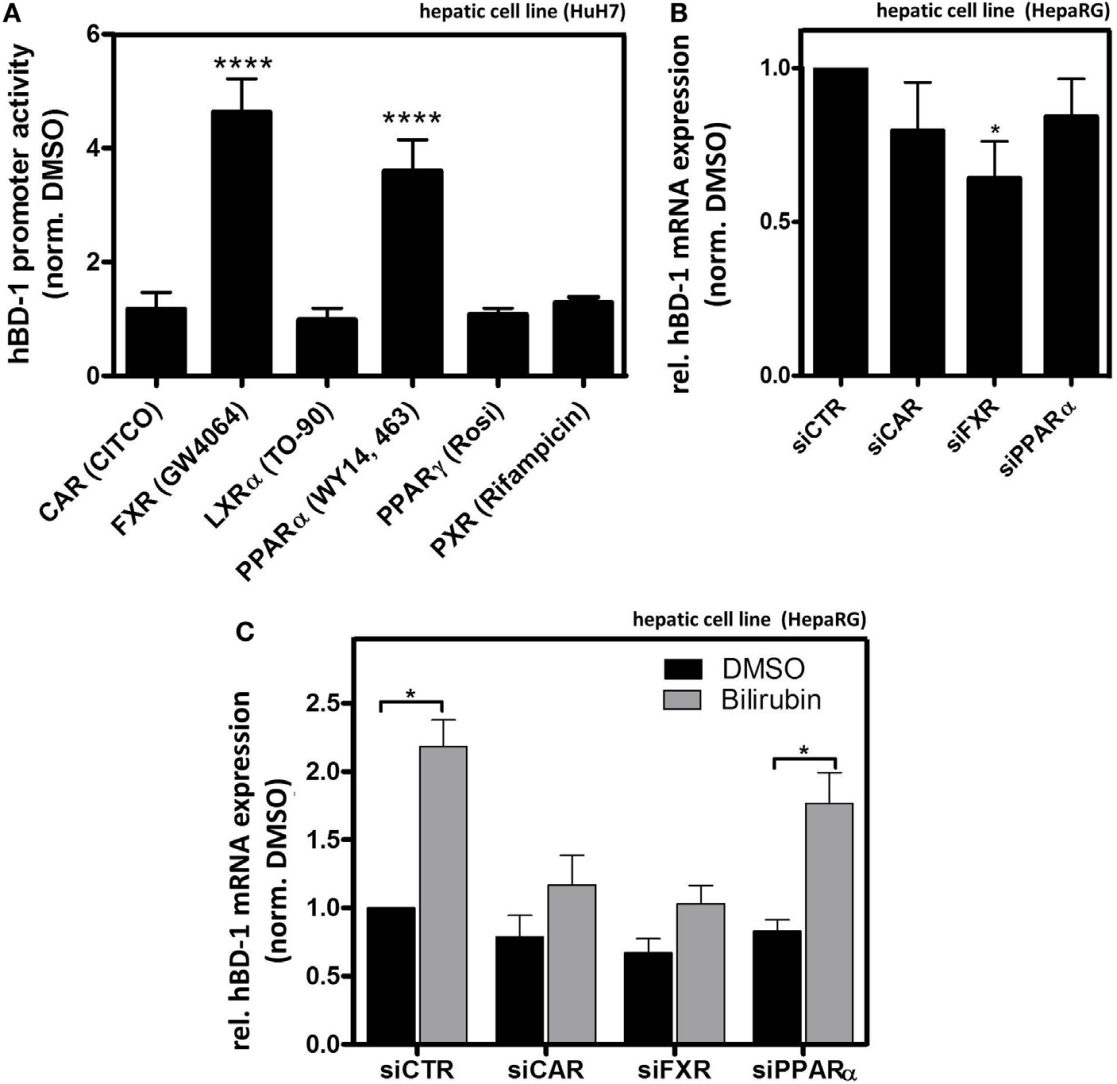
Farnesoid X receptor (FXR) and constitutive androstane receptor (CAR) modulate bilirubin-mediated hBD-1 induction. **(A)** Quantitative luciferase reporter gene assays with a construct containing 1,100 bp upstream region of hBD-1 promoter were performed in human HuH7 cells 48 h after indicated treatments with known agonists of nuclear receptors (ordered alphabetically) 5 µM CITCO (CAR), 10 µM GW4064 (FXR), 10 µM TO-90 (LXRα), 100 µM WY14,643 (PPARα), 10 µM Rosiglitazone (PPARγ, Rosi), and 10 µM of Rifampicin (PXR) in total 72 h after the transfection with the luciferase-containing constructs. The cells were co-transfected with the vector containing Renilla luciferase for normalization of transfection efficiency. The bars represent average fold change lucifearse induction normalized to solvent, DMSO, and control. Error bars indicate SD between three independent experiments. ****p < 0.001. **(B)** qRT-PCR assessment of hBD-1 mRNA expression in HepaRG lysates following transfections with the indicated small interfering RNAs (siRNAs), *p < 0.05. **(C)** qRT-PCR analysis of hBD-1 expression in HepaRG cells upon treatment with siRNAs targeting PPARα (siPPARα), FXR (siFXR), CAR (siCAR), and non-targeting scramble siRNA (siCTR) (black bars) and in combination with bilirubin treatment for 48 h (grey bars). The bars represent average fold change expression normalized to hBD-1 expression in the cells treated with the solvent, DMSO, control, and siCTR. Error bars indicate SD between three independent experiments. *p < 0.05 compared to DMSO/siCTR control condition.

To directly investigate the potential role of several NRs on hBD-1 expression we screened HuH7 cells using luciferase activity assay following transfection with an hBD-1 promoter-containing construct combined with the addition of known NR agonists to the culture medium. As shown in Figure 6A, only GW4064 activating FXR and WY14, 643 acting on PPARα out of six tested NR activators significantly induced promoter activity of hBD-1. However, since bilirubin was previously identified as an indirect activator of CAR (35), we also included CAR in the following experiments.

Consistent with the above-mentioned findings, in HepaRG cells siFXR significantly reduced basal hBD-1 expression, whereas a slight inhibition by siCAR and siPPARα was not statistically significant (Figure 6B) (for siRNA efficiency see Figure S4 in Supplementary Material). Both siCAR and siFXR blocked the induction observed in the presence of bilirubin (Figure 6C), whereas the control siRNA and siPPARα were ineffective in this regard. These findings suggest that FXR mediates constitutive expression, whereas both CAR and FXR seem to be responsible for the bilirubin-mediated induction of hBD-1expression.

## Discussion

In this study we show that human liver tissue has strong antimicrobial activity associated with the presence of the antimicrobial active hBD-1 which is ubiquitously and strongly expressed in human hepatocytes. Co-localization with its well-known activator TXN (10) and staining by a specific antibody suggests chemical reduction and activation (18) of oxidized hBD-1 by this oxidoreductase. In contrast, none of the other known β-defensins was expressed at a comparable level. Earlier studies by Harada et al. (18) focused on the role of hBD-1 in antimicrobial defense of the intrahepatic biliary tree. Similar to our findings, hBD-2 was not expressed in hepatocytes but rather in large intrahepatic bile ducts, particularly following inflammatory stimuli like cholangitis. Similarly, other antibacterials including cathelicidin were not found at significant levels in hepatocytes (16) and therefore reduced hBD-1 is a likely candidate as the key antimicrobial in human liver.

We also show that constitutively expressed hBD-1 (and TXN) is induced by cholestasis in two different patient cohorts as well as BDL mice. In human liver tissue samples and BDL mice this induction correlates with serum bilirubin levels and is gradually increasing in parallel to histological grades of cholestasis, suggesting an important role of hBD-1 in hepatic innate immune defense in this setting. Since in cholestatic diseases both bilirubin as well as BAs accumulate in the liver (36, 37) their functional role in this condition is likely as underlined by their ability to induce hBD-1 expression *in vitro*.

Incubation experiments with agonists of NRs revealed a modulation of hBD-1 expression in human hepatocyte cell lines *via* CAR and FXR, and possibly PPARα. Both CAR and FXR seem to be involved in the induction of hBD-1 expression *via* bilirubin, whereas only FXR appears to be responsible for the basal expression of hBD-1. However, PPARα is not involved in the bilirubin-mediated induction of hBD-1. The discrepancy that the direct CAR agonist CITCO did not lead to hBD-1 promotor induction in HuH7 cells and siCAR leads to blocked bilirubin-mediated induction of hBD-1 may be explained by different ways of CAR modulation by direct and indirect mechanisms (38). It is known that dependent on the agonist, NRs can regulate different patterns of target genes. Whereas CITCO belongs to the direct ligands of CAR (39), inducing its particular conformational changes and subsequent activation, bilirubin is suggested to be an indirect inducer of CAR activity (35). Therefore, we hypothesize that CAR-dependent modulation of hBD-1 induction by bilirubin is mediated *via* indirect CAR activation.

Biliary tract infections are a well-known clinical challenge during intra- and extrahepatic cholestasis leading to potentially life threatening conditions like cholangio-sepsis (20). Our results suggest a role of reduced hBD-1 to combat this challenge in cholestatic diseases.

Besides antimicrobial activity it is known that AMPs also have other functions including immunomodulatory and anti-inflammatory properties (8). Thus, the axis between bilirubin, BAs, and induced protective hBD1 might have different functional as well as pharmacological implications in cholestasis including hepatic diseases like PSC and PBC. As outlined above PPARα as well as FXR-agonists are able to induce hBD-1 expression in hepatic cell lines. On the other hand, selective activation of FXR as well as PPARα has beneficial effects on the course of cholestatic liver diseases (40–42). In particular, several fibrates (PPARα-agonists) have positive influence on liver enzymes and course of disease in patients with PBC and PSC, an effect seen with fibrate monotherapy as well as in combination with ursodeoxycholic acid (40). Additionally, administration of fenofibrate to BDL rats also exerts beneficial effects on, e.g., hepatocellular damage, hepatic portal inflammation, hepatic necrosis, as well as levels of tumor-necrosis-alpha as marker of inflammation (43). Furthermore, the new FXR agonist obeticholic acid showed positive effects in PBC and is currently tested in PSC as well as non-alcoholic steatohepatitis (42, 44, 45). Since summer 2016 it is approved by the food and drug ministration for PBC treatment in the USA (46).

Up to now these benefits are mainly attributed to the influence of PPARα- and FXR-agonists on bile acid homeostasis (40–42). Our data lead to the hypothesis that, in addition, modulation of innate immune responses *via* NR-regulated expression of hBD-1 could be in parts responsible for the positive effects of fibrates and FXR-agonists in cholestatic liver diseases by enhancing the antibacterial defense and controlling bacterial infection in cholestatic liver tissue. In agreement with this hypothesis the FXR agonist GW 4064 (as used in our cell models) shows hepatoprotective characteristics in rat models of intra- and extrahepatic cholestasis. Cholestatic rats treated with GW 4064 had lower liver enzymes, lower necrosis scores, and decreased inflammatory cell infiltration in the liver (41). Furthermore, the AMP cathelicidin LL37 has been shown to have protective functions in BDL mice (47), underlining potential beneficial effects of AMPs in cholestasis.

Taken together we could show that hBD-1 is prominently expressed by hepatocytes and induced by bilirubin and BAs. Its expression is modulated *via* the NRs FXR and CAR. Induced expression of co-localized TXN appears to activate hBD-1 *via* reduction (10, 11), therefore increasing its ability to combat the microbial challenge in the cholestatic liver. Whether modulation of hBD-1 expression, e.g., by NR-agonist treatment, is a relevant target to optimize treatment of cholestatic liver diseases merits further pre-clinical and clinical evaluation.

## Ethics Statement

The study was approved by the ethics committees of the medical faculties of the Charité, Humboldt University and the University of Tuebingen, Germany and conducted in accordance with the Declaration of Helsinki. Written informed consent was obtained from each patient. The study was approved by the ethics committee of the University Medical Center Regensburg, Germany and conducted in accordance with the Declaration of Helsinki. Written informed consent was obtained from each patient. All mice experiments were approved by the local government “Landesamt für Landwirtschaft, Lebensmittelsicherheit und Fischerei,” Mecklenburg-Vorpommern, Germany (LALLFM-V/TSD/7221.3-1.2-049/09) and performed in accordance with the German legislation on protection of animals and the National Institutes of “Health Guide for the Care and Use of Laboratory Animals” (Institute of Laboratory Animal Resources, National Research Council; NIH publication 86-23 revised 1985).

## Author Contributions

Study concept and design (1); acquisition of data (2); analysis and interpretation of data (3); drafting of the manuscript (4); critical revision of the manuscript for important intellectual content (5); statistical analysis (6); obtained funding (7); technical or material support (8); and study supervision (9). TK: study concept and design, acquisition of data, analysis and interpretation of data, drafting of the manuscript, critical revision of the manuscript for important intellectual content, statistical analysis, obtained funding, and technical or material support. MT: study concept and design, acquisition of data, analysis and interpretation of data, drafting of the manuscript, critical revision of the manuscript for important intellectual content, statistical analysis, and technical or material support. DE: study concept and design, acquisition of data, and analysis and interpretation of data. LC: acquisition of data, and analysis and interpretation of data, and critical revision of the manuscript for important intellectual content. DM-S: acquisition of data, analysis and interpretation of data, and critical revision of the manuscript for important intellectual content. KA: acquisition of data, analysis and interpretation of data, statistical analysis, and technical or material support. BV: acquisition of data, analysis and interpretation of data, statistical analysis, and technical or material support. TW: acquisition of data, analysis and interpretation of data, statistical analysis, and technical or material support. WT: acquisition of data, analysis and interpretation of data, statistical analysis, and technical or material support. RD: acquisition of data, analysis and interpretation of data, and statistical analysis. ES: critical revision of the manuscript for important intellectual content, technical or material support, and study supervision. CB: drafting of the manuscript and critical revision of the manuscript for important intellectual content. UZ: critical revision of the manuscript for important intellectual content, obtained funding, and technical or material support. NM: critical revision of the manuscript for important intellectual content, technical or material support, study supervision. JW: Study concept and design, analysis and interpretation of data, drafting of the manuscript, critical revision of the manuscript for important intellectual content, obtained funding, technical or material support, and study supervision. All authors approved the final draft submitted.

## Conflict of Interest Statement

The authors declare that the research was conducted in the absence of any commercial or financial relationships that could be construed as a potential conflict of interest.

## Supplementary Material

Figure S1 High expression of hBD-1 mRNA in primary human hepatocytes (PHH). mRNA expression of hBD-1, hBD-2, -3, and -4 was analyzed in PHH. mRNA transcript levels are measured in an amount of 500 ng RNA. Total copy numbers are depicted. Data are presented as means ± SEM of three independent experiments for each gene assay.

Figure S2 Dot-blot control of the antibody used to specifically detect the reduced form of hBD-1 by immunohistochemistry. Left panel: hBD-1 staining showing specificity for the reduced form of hBD-1. Middle panel: negative control without (w/o) primary antibody (1°). Right panel: negative control without (w/o) secondary antibody (2°).

Figure S3 No induced expression of hBD-1 mRNA in human liver tissue with “mild” cholestasis. **(A)** mRNA expression of hBD-1 and human oxidoreductases TXN **(B)** and GRX **(C)** were analyzed in human liver tissue (*n* = 137). Control group (*n* = 87) without cholestasis was compared with “mild cholestasis” samples (alkaline phosphatase and gamma-glutamyl transferase elevation). mRNA transcript levels are measured in an amount of 20 ng cDNA. Total copy numbers are depicted. Data are presented as means ± SEM. Values between groups were considered statistically significant with *p* < 0.05.

Figure S4 Efficiency of small interfering RNAs (siRNA) knock-down in hepatic cell lines. For testing the knock-down efficiency, siRNAs targeting PPARα **(A)**, farnesoid X receptor **(B)**, and constitutive androstane receptor (CAR) **(C)** were transfected in HepaRG cells and 48 h later the lysates were analyzed for the protein level **(A,B)** or mRNA expression of the respective CAR target gene, CYP2B6 **(C)**. Exemplified western blot analysis is shown on the top **(A,B)** and the average of three independent experiments is shown in the diagram of mRNA expression **(C)**. *****p* < 0.001, ***p* < 0.01, and **p* < 0.05.

Table S1 Characteristics of hepatocyte donors for experiments with primary human hepatocytes.

Table S2 Oligonucleotide primer pairs used for qRT-PCR.
