# Supplementary figures and images for: β-Defensin 1 Is Prominent in the Liver and Induced During Cholestasis by Bilirubin and Bile Acids via Farnesoid X Receptor and Constitutive Androstane Receptor
Source: Front Immunol. 2018 Jul 27;9:1735. doi: 10.3389/fimmu.2018.01735 (PMC6072844; doi:10.3389/fimmu.2018.01735)

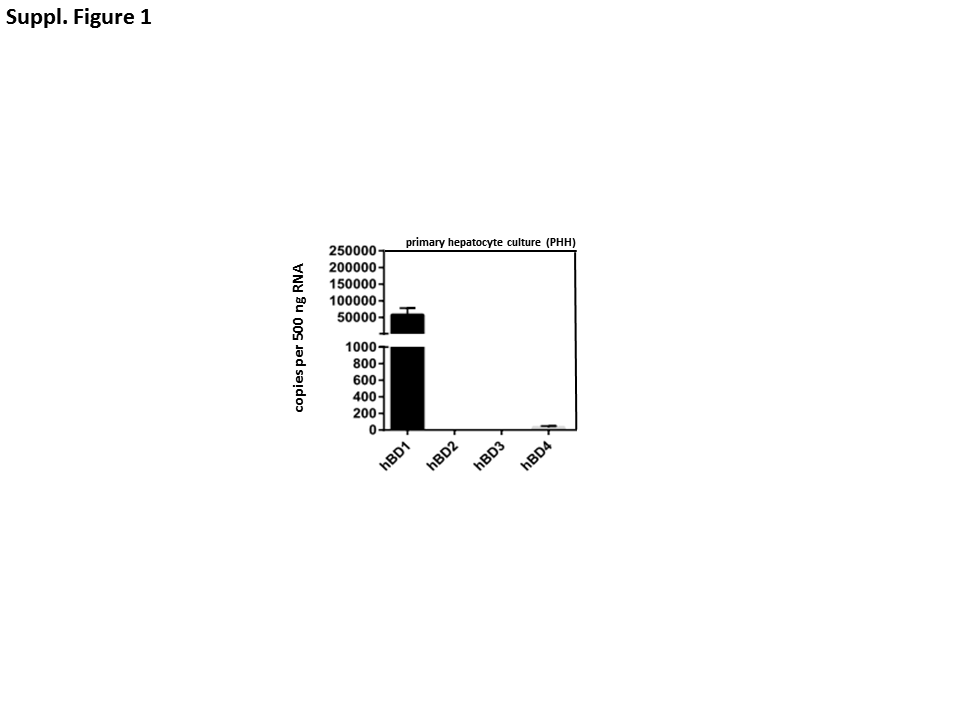

Supplement: Figure S1 — High expression of hBD-1 mRNA in primary human hepatocytes (PHH). mRNA expression of hBD-1, hBD-2, -3, and -4 was analyzed in PHH. mRNA transcript levels are measured in an amount of 500 ng RNA. Total copy numbers are depicted. Data are presented as means ± SEM of three independent experiments for each gene assay. [file image_1.tif]

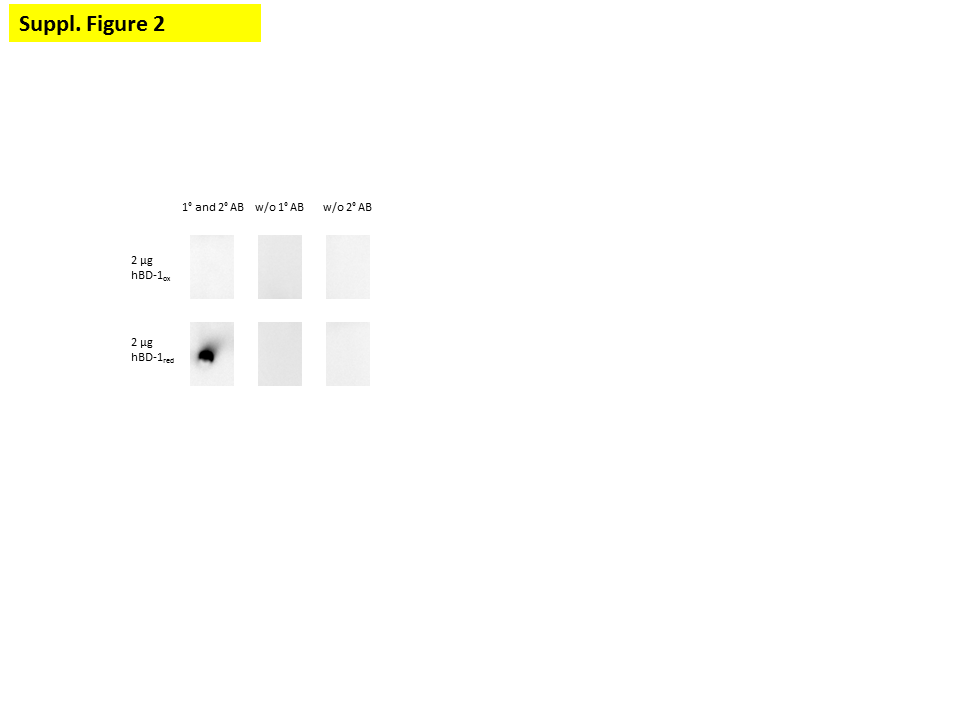

Supplement: Figure S2 — Dot-blot control of the antibody used to specifically detect the reduced form of hBD-1 by immunohistochemistry. Left panel: hBD-1 staining showing specificity for the reduced form of hBD-1. Middle panel: negative control without (w/o) primary antibody (1°). Right panel: negative control without (w/o) secondary antibody (2°). [file image_2.tif]

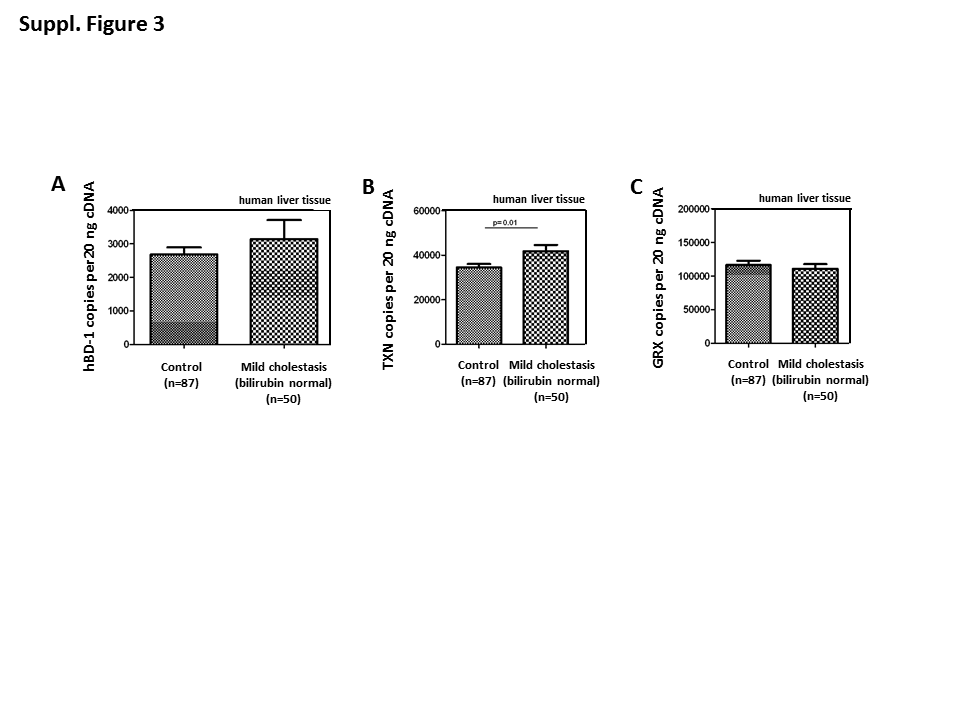

Supplement: Figure S3 — No induced expression of hBD-1 mRNA in human liver tissue with “mild” cholestasis. (A) mRNA expression of hBD-1 and human oxidoreductases TXN (B) and GRX (C) were analyzed in human liver tissue (n = 137). Control group (n = 87) without cholestasis was compared with “mild cholestasis” samples (alkaline phosphatase and gamma-glutamyl transferase elevation). mRNA transcript levels are measured in an amount of 20 ng cDNA. Total copy numbers are depicted. Data are presented as means ± SEM. Values between groups were considered statistically significant with p < 0.05. [file image_3.tif]

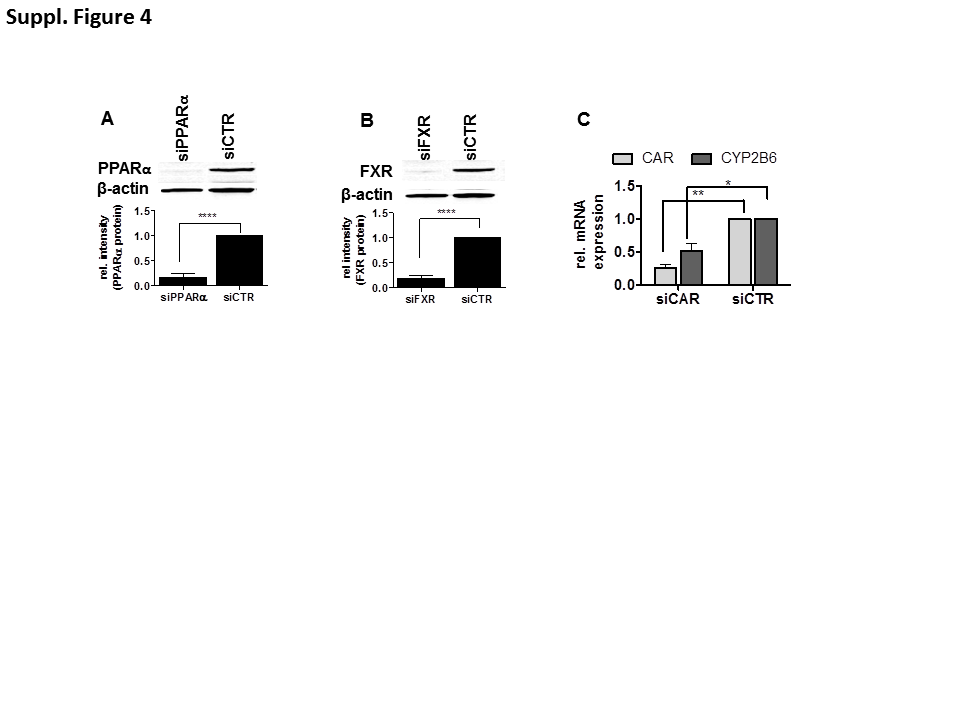

Supplement: Figure S4 — Efficiency of small interfering RNAs (siRNA) knock-down in hepatic cell lines. For testing the knock-down efficiency, siRNAs targeting PPARα (A), farnesoid X receptor (B), and constitutive androstane receptor (CAR) (C) were transfected in HepaRG cells and 48 h later the lysates were analyzed for the protein level (A,B) or mRNA expression of the respective CAR target gene, CYP2B6 (C). Exemplified western blot analysis is shown on the top (A,B) and the average of three independent experiments is shown in the diagram of mRNA expression (C). ****p < 0.001, **p < 0.01, and *p < 0.05. [file image_4.tif]
