# Supplementary material for: β-Defensin 1 Is Prominent in the Liver and Induced During Cholestasis by Bilirubin and Bile Acids via Farnesoid X Receptor and Constitutive Androstane Receptor
Source: Front Immunol. 2018 Jul 27;9:1735. doi: 10.3389/fimmu.2018.01735 (PMC6072844; doi:10.3389/fimmu.2018.01735)
Supplement: Table S1 — Characteristics of hepatocyte donors for experiments with primary human hepatocytes. [file table_1.docx]

| **Donor** | **Age (years)** | **Sex** | **Pathology (indication of liver resection)** |
| --- | --- | --- | --- |
| 1 | 65 | female | liver metastasis of colorectal cancer |
| 2 | 67 | female | liver metastasis of colorectal cancer |
| 3 | 59 | female | liver metastasis due to cancer of unknown primary |
| 4 | 47 | female | liver metastasis due to a neuroendocrine tumor |
| 5 | 50 | male | liver metastasis of colorectal cancer |
| 6 | 51 | female | liver metastasis of renal cancer |
| 7 | 51 | female | liver metastasis of colorectal cancer |

**Suppl. table 1**
