# Supplementary material for: β-Defensin 1 Is Prominent in the Liver and Induced During Cholestasis by Bilirubin and Bile Acids via Farnesoid X Receptor and Constitutive Androstane Receptor
Source: Front Immunol. 2018 Jul 27;9:1735. doi: 10.3389/fimmu.2018.01735 (PMC6072844; doi:10.3389/fimmu.2018.01735)
Supplement: Table S2 — Oligonucleotide primer pairs used for qRT-PCR. [file table_2.docx]

**Suppl. table 2**

| **Product** | **Forward primer (5`->3`)** | **Reverse primer (5`->3`)** |
| --- | --- | --- |
| hBD-1 | GGC CTC AGG TGG TAA CTT TCT | TTC TTC TGG TCA CTC CCA GC |
| hBD-2 | ATC AGC CAT CAG GGT CTT GT | GAG ACC ACA GGT GCC AAT TT |
| hBD-3 | TGA AGC CTA GCA GCT ATG AGG ATC | CCG CCT CTG ACT CTG CAA TAA |
| hBD-4 | AGA TCT TCC AGT GAG AAG CGA | GAC ATT TCT TCC GGC AAC GG |
